# Identification of druggable small molecule antagonists of the *Plasmodium falciparum* hexose transporter PfHT and assessment of ligand access to the glucose permeation pathway via FLAG-mediated protein engineering

**DOI:** 10.1371/journal.pone.0216457

**Published:** 2019-05-09

**Authors:** Monique R. Heitmeier, Richard C. Hresko, Rachel L. Edwards, Michael J. Prinsen, Ma Xenia G. Ilagan, Audrey R. Odom John, Paul W. Hruz

**Affiliations:** 1 Department of Pediatrics, Washington University School of Medicine, St Louis, MO, United States of America; 2 High Throughput Screening Center, Washington University School of Medicine, St Louis, MO, United States of America; 3 Department of Molecular Microbiology, Washington University School of Medicine, St Louis, MO, United States of America; 4 Department of Cell Biology and Physiology, Washington University School of Medicine, St Louis, MO, United States of America; University of Melbourne, AUSTRALIA

## Abstract

Although the *Plasmodium falciparum* hexose transporter PfHT has emerged as a promising target for anti-malarial therapy, previously identified small-molecule inhibitors have lacked promising drug-like structural features necessary for development as clinical therapeutics. Taking advantage of emerging insight into structure/function relationships in homologous facilitative hexose transporters and our novel high throughput screening platform, we investigated the ability of compounds satisfying Lipinksi rules for drug likeness to directly interact and inhibit PfHT. The Maybridge HitFinder chemical library was interrogated by searching for compounds that reduce intracellular glucose by >40% at 10 μM. Testing of initial hits via measurement of 2-deoxyglucose (2-DG) uptake in PfHT over-expressing cell lines identified 6 structurally unique glucose transport inhibitors. WU-1 (3-(2,6-dichlorophenyl)-5-methyl-N-[2-(4-methylbenzenesulfonyl)ethyl]-1,2-oxazole-4-carboxamide) blocked 2-DG uptake (IC_50_ = 5.8 ± 0.6 μM) with minimal effect on the human orthologue class I (GLUTs 1–4), class II (GLUT8) and class III (GLUT5) facilitative glucose transporters. WU-1 showed comparable potency in blocking 2-DG uptake in freed parasites and inhibiting parasite growth, with an IC_50_ of 6.1 ± 0.8 μM and EC_50_ of 5.5 ± 0.6 μM, respectively. WU-1 also directly competed for N-[2-[2-[2-[(N-biotinylcaproylamino)ethoxy)ethoxyl]-4-[2-(trifluoromethyl)-3H-diazirin-3-yl]benzoyl]-1,3-bis(mannopyranosyl-4-yloxy)-2-propylamine (ATB-BMPA) binding and inhibited the transport of D-glucose with an IC_50_ of 5.9 ± 0.8 μM in liposomes containing purified PfHT. Kinetic analysis revealed that WU-1 acts as a non-competitive inhibitor of zero-trans D-fructose uptake. Decreased potency for WU-1 and the known endofacial ligand cytochalasin B was observed when PfHT was engineered to contain an N-terminal FLAG tag. This modification resulted in a concomitant increase in affinity for 4,6-O-ethylidene-α-D-glucose, an exofacially directed transport antagonist, but did not alter the K_m_ for 2-DG. Taken together, these data are consistent with a model in which WU-1 binds preferentially to the transporter in an inward open conformation and support the feasibility of developing potent and selective PfHT antagonists as a novel class of anti-malarial drugs.

## Introduction

Despite intensive efforts to control the spread of infection with *Plasmodium* species, the causative agent of malaria, disease prevalence remains alarmingly high, with over 219 million new cases world-wide in 2017 alone [1]. While substantial investment of monetary and intellectual resources to combat malaria has resulted in a 93% decline in mortality over the past 5 years, over 435,000 deaths still occur annually, mostly in children [1]. The emergence of parasite resistance to all available therapeutics including delayed clearance to artemisinin- based compounds has hindered efforts to eradicate this devastating disease [2]. Thus, there is an ongoing need to develop novel anti-malarial agents with high potency, low production cost, sustained efficacy within disease endemic areas, and favorable pharmacokinetic profiles that allow single dose treatment regimens [3]. With recent advances in understanding the molecular mechanisms responsible for parasite replication, new avenues have emerged for the design and implementation of effective mitigation strategies [4, 5].

One highly promising approach is to directly interfere with parasite hexose transport. Glucose is the primary source of energy needed by blood-stage parasites for biomass production and ATP synthesis. The malarial glucose transporter, *Plasmodium falciparum* hexose transporter (PfHT), first identified by Woodrow *et al*. in 1999 [6] is essential for parasite survival [7]. Chemical validation of PfHT as an antimalarial target was accomplished with the discovery of compound 3361, a glucose analog, as an antagonist of *Plasmodium* hexose transporter [8, 9]. Compound 3361 is highly selective for PfHT over the human orthologue GLUT1 and also inhibits asexual intra-erythrocytic growth in culture [8]. Compound 3361 is also active against *P*. *berghei* liver and transmission stage parasites, suggesting that PfHT may have highly desirable full life cycle activity [8, 10]. However, while 3361 validates efforts to target PfHT, this compound is not itself considered drug-like and is therefore not a viable candidate for lead development [9].

GLUT1 activity is also important for glucose uptake into red blood cells and hepatocytes and GLUT1-mediated uptake has been shown to be enhanced in *Plasmodium berghei*-infected cells [11]. Inhibition of GLUT1 under these circumstances may be beneficial for inhibiting *P*.*berghei* infection. However, due to the ubiquitous expression of this transporter and its critical role in maintaining basal glucose delivery to tissues, inhibition of GLUT1 would be expected to result in significant host toxicity.

Initial efforts to screen for additional small molecule PfHT antagonists was performed in hexose transport null mutants of *Leishmania mexacana* parasites engineered to heterologously express the *Plasmodium falciparum* transporter [12]. This approach yielded several bioactive compounds with varying potency and selectivity for PfHT over the mammalian transporter GLUT1. The physical and pharmacokinetic profile of these pre-lead hits are not sufficient for use as an orally bioavailable drug and will require extensive structure-activity analysis for optimization [12].

Our identification of the clinically marketed drug lopinavir, an HIV protease inhibitor, as a direct antagonist of PfHT further advanced this pharmacological approach to block parasite replication. However, lopinavir has a relatively poor potency (16 μM IC_50_) against blood stage parasites and shows higher selectivity for the human insulin-responsive glucose transporter GLUT4 over PfHT [13]. Drugs that block GLUT4 activity induce insulin resistance [14]. Therefore, the development of novel therapeutics targeting PfHT requires identification of compounds with substantially improved potency and selectivity over the human GLUT orthologues.

To facilitate the identification of novel chemical scaffolds with potent inhibitory activity toward PfHT, we developed a robust high-throughput screening assay that allows real-time detection of intracellular glucose levels in cell lines engineered to selectively over-express a single facilitative glucose transporter. This assay has been validated by screening of the MMV Malaria Box of 400 compounds with known anti-malarial activity [15]. Similar to compound 3361, the novel hits from this screen do not possess sufficient drug-like structure for pharmaceutical development. We therefore sought to begin with a chemical library specifically designed to follow Lipinski’s rules for potential as an orally deliverable drug. The Maybridge HitFinder collection, consisting of 14,399 small molecules with drug-like diversity and chemical stability, provided a suitable screening platform. We report here the identification of several novel chemical scaffolds for hit-to-lead optimization and characterization of both potency and selectivity for PfHT over human glucose transporters.

With the further goal of developing drugs that will maintain efficacy during widespread clinical use, we have characterized the physical interaction of inhibitory compounds with the transporter. Given that hexose transport is essential for parasite survival, we hypothesized that compounds that bind to PfHT within the glucose permeation pathway will be less susceptible to resistance, since mutations that reduce drug affinity would be more likely to also impair transporter function. We here provide evidence for structural interactions between WU-1 and PfHT protein in light of the current rocker-switch model for transporter conformational dynamics [16]. As discussed in detail in this report, these data can facilitate the development of novel antimalarial drugs that are less susceptible to parasite resistance through mutational change. This information also provides novel insight on the conformational dynamics of the family of structurally related facilitative hexose transporters.

## Materials and methods

### Materials

WU-1 (3-(2,6-dichlorophenyl)-5-methyl-N-[2-(4-methylbenzenesulfonyl)ethyl]-1,2-oxazole-4-carboxamide, cat# 002-890-346), WU-2 (3-(3-bromo-2,6-dimethoxy-5-nitrobenzoyl)-1-(2-chloro-6-methylphenyl)urea, cat# 002-892-269), WU-3 (2-amino-5-phenyl-N'-[(E)-(3,4,5-trimethoxyphenyl)methylidene]thiophene-3-carbohydrazide, cat# 002-903-252 and WU-5 ((3E,5E)-3,5-bis[(3,4,5-trimethoxyphenyl)methylidene]oxan-4-one, cat# 001-840-877) were purchased from MolPort, Riga, Latvia. [^14^C]2-deoxyglucose ([^14^C]2-DOG), [^3^H]2-DG (2-DG), [^3^H]D-fructose (D-Frc), [^3^H]-D-glucose, and [^3^H]-L-glucose were purchased from American Radiolabels Inc. (St. Louis, MO). GLUT1 short hairpin RNA (shRNA) was obtained through the RNA interference (RNAi) core at Washington University School of Medicine. HEK293 cells were acquired from the American Type Culture Collection (ATCC). Egg phosphatidylcholine (eggPC), palmitoyloleoyl phosphatidylethanolamine (POPE), and palmitoyloleoyl phosphatidic acid (POPA) were purchased from Avanti Polar Lipids Inc. (Alabaster, AL). Lauryl maltose neopentyl glycol (LMNG) detergent was obtained from Anatrace (Maumee, OH). PEG-biotin cap-ATB-BMPA was purchased from Toronto Research Chemicals, Inc. (Ontario, Canada). FLAG tag rabbit antibody (2368) was obtained from Cell Signaling Technology (Danvers, MA). All other reagents were purchased from Sigma or as otherwise indicated.

### PfHT antibodies

Rabbit antisera raised against a synthetic PfHT carboxy-terminal peptide (amino acids 481–496 (C-GGEIGTSPYITMEERQ)) were prepared by ABclonal Technology (Woburn,MA) and used in the immunoblot analyses.

### Cell line generation

Generation of HEK293 cells overexpressing PfHT and human GLUTs 1–4 were described elsewhere [13, 15]. Briefly, HEK293 cells were transfected with pcDNA3.1 FLII12Pglu-7006 (Addgene), containing the fluorescence resonance energy transfer (FRET) glucose sensor (HEK293-FLIP) and PfHT, human GLUT1 (hGLUT1), hGLUT2, hGLUT3, hGLUT4, hGLUT5 or hGLUT8 using Optifect (Life Technologies) according to manufacturer’s specifications. To increase retention of GLUT8 at the plasma membrane, the dileucine sorting signal E^7^E^8^XXXLL was modified to R^7^R^8^XXXLL as described in [17]. Stable transfectants were selected for G418 and hygromycin resistance to select for FRET sensor and hexose transporter, respectively. HEK293-FLIP cells were also used to generate cells stably transfected with FLAG-tagged PfHT (FTPfHT). FLAG tag (DYKDDDDK) followed by a triple glycine linker and a tobacco etch virus (TEV) protease site (ENLYFQS) followed by a triple glycine linker was added to the N-terminus of PfHT via PCR [18] using Phusion Hot Start II High-Fidelity DNA Polymerase (Fisher Scientific). Single clones were selected by comparing their abilities to transport glucose as assayed via FRET and radiolabeled hexose uptake. In all cell lines, except for HEK293 cells overexpressing hGLUT1 and hGLUT5, native hGLUT1 was knocked down using shRNA as described elsewhere [13]. Transcript levels of the individual GLUTs expressed in each cell line are as described in [13, 15] and in S1 Fig.

### High-throughput screening

Black opaque 384-well plates (Greiner Bio-One) were pretreated with 25 μg/ml of polyethylenimine (PEI; 750 kDa; Sigma-Aldrich) solution containing 150 mM NaCl to maintain cell adhesion during washing steps. After 20 min of incubation at room temperature, PEI was aspirated and wells were air dried for 5 min. Using a Multidrop384 dispenser (Thermo Fisher Scientific), PfHT-FLIP cells were plated at 20,000 cells/well. After cell plating, plates were left in the sterile hood at room temperature for 45 min before transfer to the incubator which helped reduce edge effects [19]. Compound screening was performed the following day using the integrated and automated screening system (Beckman Coulter) at the Washington University High Throughput Screening Center. SAMI EX software (Beckman Coulter) was used to design and execute the screening protocol. Maybridge HitFinder library compounds were pre-dispensed into 384-well Greiner polypropylene plates (Cat# 781280) using the Hummingbird XL. Using a Multidrop Combi dispenser, the compounds were prediluted in glucose-free HEPES-buffered saline solution (HBSS) (146 mM NaCl, 4.7 mM KCl, 0.6 mM MgSO_4_, 1.6 mM NaHCO_3_, 0.13 mM NaH_2_PO_4_, 2.5 mM CaCl_2_, 20 mM HEPES [pH 7.3]). We prepared a 1:900 dilution of 10 mM stock solution of the compounds for an 11.11 μM final concentration. DMSO (0.11% DMSO) and cytochalasin B (CB, 200 μM) were used as negative and positive controls for inhibition, respectively. To initiate the screening assay, cells were washed twice with 50 μl of HBSS per well using an ELx405 plate washer (Biotek), then incubated for 30 minutes (starvation period) after addition of 45 μl of diluted compound using the BiomekFX Liquid Handler (Beckman Coulter). To initiate the uptake, 5 μl of 50 mM glucose was added to the wells (final compound concentration, 10 μM; final glucose concentration, 5 mM) using a Multidrop384 dispenser (Thermo Fisher Scientific). After incubation at room temperature for 120 min, fluorescence was measured using a 2102 EnVision multilabel plate reader (PerkinElmer) at an excitation wavelength of 436 nm (cyan fluorescent protein [CFP]) and emission wavelengths of 485 nm (CFP) and 535 nm (yellow fluorescent protein [YFP]). % inhibition was determined using the plate controls by subtracting the YFP/CFP ratio (FRET ratio) for CB controls from that of the vehicle (DMSO) controls and then calculating the % decrease in signal for each single point compound tested.

### *P*. *falciparum* culture

*P*. *falciparum* strain 3D7 was obtained from the Malaria Research and Reference Reagent Resource Center (MR4, ATCC, Manassas, VA). Parasites were cultured in a 2% suspension of human erythrocytes and RPMI 1640 (Sigma) medium supplemented with 27 mM sodium bicarbonate, 11 mM glucose, 5 mM HEPES, 1 mM sodium pyruvate, 0.37 mM hypoxanthine, 0.01 mM thymidine, 10 μg/ml gentamicin, and 0.5% Albumax (Gibco) at 37°C, 5% O_2_/ 5% CO_2_ / 90% N_2_ atmosphere as previously described [20, 21].

### *P*. *falciparum* growth inhibition assays

Asynchronous *P*. *falciparum* cultures were diluted to 1% parasitemia and were treated with compounds at concentrations ranging from 0.195 μM—100 μM. Growth inhibition assays were performed in 100 μl cultures in opaque 96-well plates. Parasite growth was quantified after 3 days by measuring DNA content using Picogreen (Life Technologies)[22]. Fluorescence was measured by a FLUOstar Omega microplate reader (BMG Labtech) at 485 nm excitation and 528 nm emission. IC_50_ values were calculated by least squares nonlinear regression analysis using GraphPad Prism 6.0 software.

### *P*. *falciparum* hexose uptake

Glucose uptake by *P*. *falciparum* was performed essentially as described [23]. *P*. *falciparum* strain 3D7 was cultured in 100 mm tissue culture dishes (Techno Plastic Products) in a 2% suspension of human erythrocytes and RPMI 1640 medium until reaching > 7.5% parasitemia. Cells were pelleted via centrifugation and resuspended in RPMI 1640 medium. To determine the uptake of radiolabeled glucose into the parasite, parasites were isolated from infected erythrocytes by permeablization with saponin (0.05 w/v), followed by centrifugation, aspiration of supernatant and washing of the pellet 3X with saline solution (135 mM NaCl, 2 mM KCl, 25 mM HEPES, 0.2 mM glucose and 1 mM MgCl_2_; pH 7.1) to remove cellular debris. WU-1 or DMSO vehicle were added to 100 μl parasites (resuspended in saline) 5 min prior to the addition of 100 μl [^14^C]2-DOG (0.2- μCi/ml final concentration). 2-DG uptake was stopped after 2 min by layering the entire reaction over 300 μl of an oil mixture (5:4 mix of dibutyl phthalate / dinonyl phthalate) in Eppendorf tubes and centrifuged at 15,850 × *g* for 2 mins. The ratio of the estimated intracellular concentration of radiolabel relative to the extracellular concentration were calculated as described [23]. Uptake of radiolabeled hexose into HEK293 cells was measured in glucose-free HBSS as described previously [24]. Briefly, cells were plated at 400,000 cells/well overnight at 37°C in 24-well plates pre-treated with PEI. After 30-min incubation at 37°C in glucose-free HBSS in the presence of vehicle or increasing concentrations of compound, 2-DG uptake was measured in glucose-free HBSS for 4 min at 37°C. Cultures were washed in ice-cold 1X PBS and lysed in 0.1% Triton X-100 in 1X PBS. Radiolabeled hexose uptake was determined by liquid scintillation counting (Ultima Gold, Thermo Fisher) and normalized to total protein per well. Uptake data are plotted as percent uptake relative to vehicle-treated cells. Kinetic analyses were performed using the indicated concentrations of substrate and WU-1, with quenching of uptake after 1 min incubation. Incubation times had been previously determined as being in the linear range of time-dependent radiolabeled hexose uptake (2 min for 2-DG uptake into freed parasites; 2 and 4 min for D-Frc and 2-DG uptake HEK cell lines, respectively). Data were fit by least squares nonlinear regression analysis using GraphPad Prism 6.0 software.

### Purification of FTPfHT

SalI and NotI restriction sites were introduced to the 5’ and 3’ ends, respectively, of FTPfHT cDNA via PCR and then this construct was placed into the multiple cloning site of the pACMV-tetO vector [25]. Stable tetracycline-inducible HEK293S GnTI cell lines expressing FTPfHT were generated using the same protocol previously described for FTGLUT4 [26]. FTPfHT protein was solubilized from FTPfHT/HEK293S GnTI cell pellets with lauryl maltose neopentyl glycol (LMNG) detergent and purified using M2 anti-FLAG affinity gel (Sigma) as described in [27]. The FLAG tag was cleaved using tobacco etch virus (TEV) protease (plasmid purchased from Addgene, Cambridge, MA) at a ratio of 1:20 TEV/PfHT overnight at 4°C in the presence of 1 mM DTT. Based on SDS-PAGE and Blue BANDit protein stain (Amresco, Solon, OH), PfHT protein after FLAG elution was ~95% pure.

### PfHT liposome reconstitution and [^3^H]-glucose uptake

Liposomes containing PfHT were made according to Hresko et al. [27]. Briefly, purified PfHT protein in LMNG micelles described above were mixed with Triton X-100 destabilized eggPC/POPA/POPE (70:15:15) large unilamellar vesicles (lipid to protein ratio of 100–125:1 (w/w)). By slowly removing the detergents with Amberlite XAD-2 beads (Supelco Analytical, Bellefonte, PA), PfHT protein spontaneously inserts into the liposomes. After removal of the beads, liposomes were collected by ultracentrifugation, resuspended in buffer, and flash-frozen with liquid nitrogen. Frozen liposomes containing purified PfHT protein were thawed at room temperature followed by extrusion through a 200-nm polycarbonate filter to create liposomes of uniform size. Uptake was started by adding [^3^H]-D-glucose (1 μCi/ml, 200 μM cold D-glucose) to PfHT proteoliposomes at room temperature. Transport was stopped at different times with 5 ml of ice-cold quench buffer (50 mM potassium phosphate, 130 mM KCl, pH 7.4) and then filtered by vacuum on a 0.2-μm mixed cellulose ester filter (Advantec, Durham, NC). Filters were subsequently washed with an additional 10 ml of quench buffer. Scintillation fluid (Filter Count, PerkinElmer, Waltham, MA) was added to the filters and the radioactivity counted. [^3^H]-L-glucose (1 μCi/ml, 200 μM cold L-glucose) was used to determine nonspecific transport. Specific uptake was calculated by subtracting nonspecific [^3^H]-L-glucose from [^3^H]-D-glucose uptake. Uptake was normalized to the amount of transporter per reaction, determined by analyzing aliquots of transporter-containing liposomes along with a BSA standard curve on an SDS-polyacrylamide gel stained with Blue Bandit protein stain and quantified using an Odyssey infrared imaging system (LI-COR Biosciences, Lincoln, NB). Data were fitted by least squares nonlinear regression analysis using GraphPad Prism 6.0.

### Photolabeling of PfHT liposomes

WU-1 inhibitor was added to PfHT liposomes for 10 min at room temperature. Samples (final volume, 110 μl) were then incubated for 10 min at room temperature with biotinylated ATB-BMPA (50 μM final concentration) and then placed on ice prior to UV irradiation. Reactions were transferred to a 24-well low protein retention culture dish (Costar, Corning, NY) and then irradiated at room temperature 5 cm from a Green Spot UV lamp for 1 min (30 sec of light followed by 30 sec of darkness followed by 30 sec of light). Unbound ATB-BMPA was removed using a 0.5 ml Zeba Spin Column (Thermo Scientific, Rockford, IL). Samples were analyzed by immunoblot analysis using a fluorescently labeled streptavidin (LI-COR Biosciences, Lincoln, NE). The amount of PfHT protein in each sample was quantified by SDS-PAGE stained with Blue BANDIT protein stain using a BSA standard curve. The streptavidin signal was then normalized to the amount of PfHT protein.

### Statistics

Statistical analysis between groups was performed using paired student’s t-test as indicated in Results and Figure Legends.

## Results

### Screening assay

Our aim was to identify potent and selective inhibitors of *P*. *falciparum* hexose transporter, PfHT. Using our novel FRET-based high-throughput HEK293PfHT screening assay [15], we screened the Maybridge HitFinder library of 14,399 compounds. This assay was optimized for high-throughput application in a 384-well format using the Z’ factor and the coefficient of variation (CV) as measures of assay robustness [28]. Dimethylsulfoxide (DMSO) and cytochalasin B (CB) were used as negative and positive controls for inhibition of glucose uptake (inhibition of FRET signal), respectively. We were able to routinely obtain a Z’ factor of ~0.8 (a perfect assay would have a Z’ factor of 1.0) and a CV of ~2%. Compounds were screened at a concentration of 10 μM for the ability to decrease the FRET ratio by more than 40%. We identified 407 compounds (2.8% hit rate) as potential hits for further analysis. Of these, 212 compounds were intrinsically fluorescent potential “false positives” and were removed from further consideration. The remaining 195 compounds were tested for glucose uptake inhibition via single point determination of inhibition of radiolabeled 2-deoxy-glucose (2-DG) uptake assay using PfHT-overexpressing HEK293 cells. 6 of the 195 potential hits, inhibited 2-DG uptake in PfHT-overexpressing HEK293 cells (> 40%) which translates to a confirmed hit rate of 0.042%.

### Confirmation of hits using 2-DG uptake

**Fig 1:**
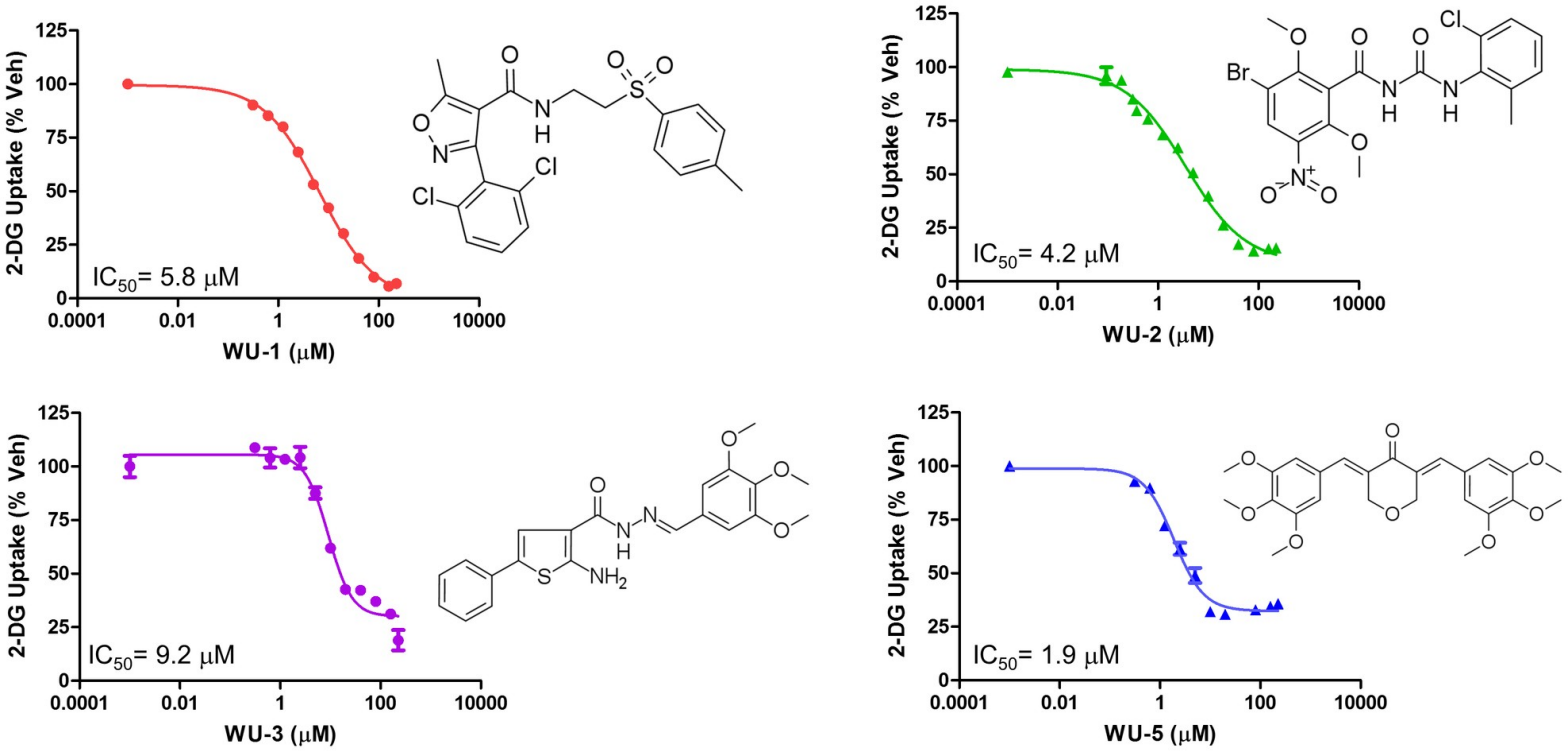
IC_50_ determination of glucose uptake by PfHT-overexpressing HEK293 cells. Uptake of 2-DG by HEK293 cells overexpressing PfHT at increasing concentrations of the indicated compounds. IC_50_s were calculated using nonlinear regression analysis IC_50_ determinations using GraphPad Prism 6.0. Uptake data are expressed as means ± SEMs (n = 3). Chemical structures of the compounds tested are shown for comparison.

Of the 6 confirmed hits, 4 compounds, “WU-1”, “WU-2”, “WU-3”, and “WU-5”, were commercially available and acquired for further characterization. We determined the potency of each compound to inhibit PfHT-mediated glucose uptake by 2-DG uptake IC_50_ determinations. As shown in Fig 1, all four compounds inhibit PfHT with the order of inhibition WU-5 (1.9 μM) > WU-2 (4.2 μM) > WU-1 (5.8 μM) > WU-3 (9.2 μM).

### Isoform selectivity determination, growth inhibition of P. falciparum 3D7, and inhibition of glucose uptake in freed parasites

**Fig 2:**
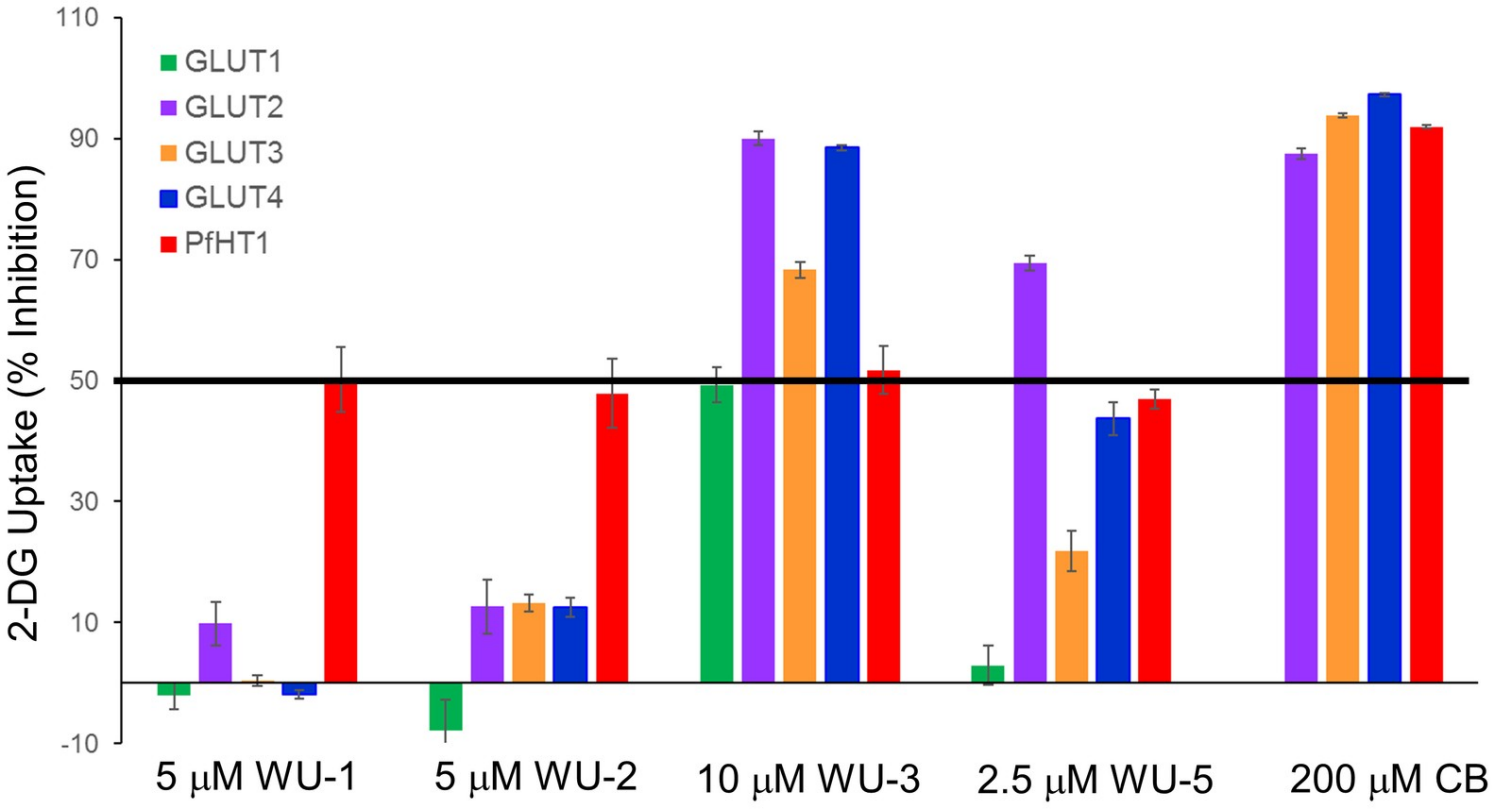
Specificity of hit compounds for PfHT over class I human orthologues. Uptake of 2-DG by HEK293 cells overexpressing hGLUT1-4 in the presence of the indicated compounds at indicated concentrations approximating their IC_50_ for PfHT as shown in Fig 1. CB was used as a positive control.

To mitigate potential off-target effects of candidate PfHT antagonists on human glucose transporter orthologues, we used our novel HEK293 cell lines that were engineered to selectively over-express single GLUT isoforms. As shown in S1 Fig and references [13] and [15], quantitative PCR confirmed the desired expression of the single desired GLUT isoform over endogenous hexose transporters. We further determined that 2DG transport activity was at least 3-11-fold above the untransfected native HEK293 cell line (S2 Fig). This likely represents a lower limit of exogenous transporter contribution to glucose uptake, since it has previously been shown that over-expression of exogenous GLUTs in this cell line results in suppression of endogenous transporter expression [29]. Using the IC_50_ concentration of each compound for the inhibition of PfHT, we determined the isoform selectivity of these compounds for PfHT vs. human GLUTs. As shown in Fig 2, compound WU-1 exhibited good selectivity for PfHT over the class I GLUTs, inhibiting only GLUT2 by ~10%. WU-2 was somewhat less selective, inhibiting GLUTs -2, -3 and -4 by ~10–12%. Compound WU-3 was non-selective, inhibiting all the class I GLUTs by 50% or more at the PfHT IC_50_ concentration. WU-5 did not inhibit GLUT1, but significantly inhibited GLUT2 (~70%), GLUT3 (~20%) and GLUT4 (~45%). Due to non-selectivity, characterization of compounds WU-3 and WU-5 was not pursued further and we continued with compounds WU-1 and WU-2.

**Fig 3:**
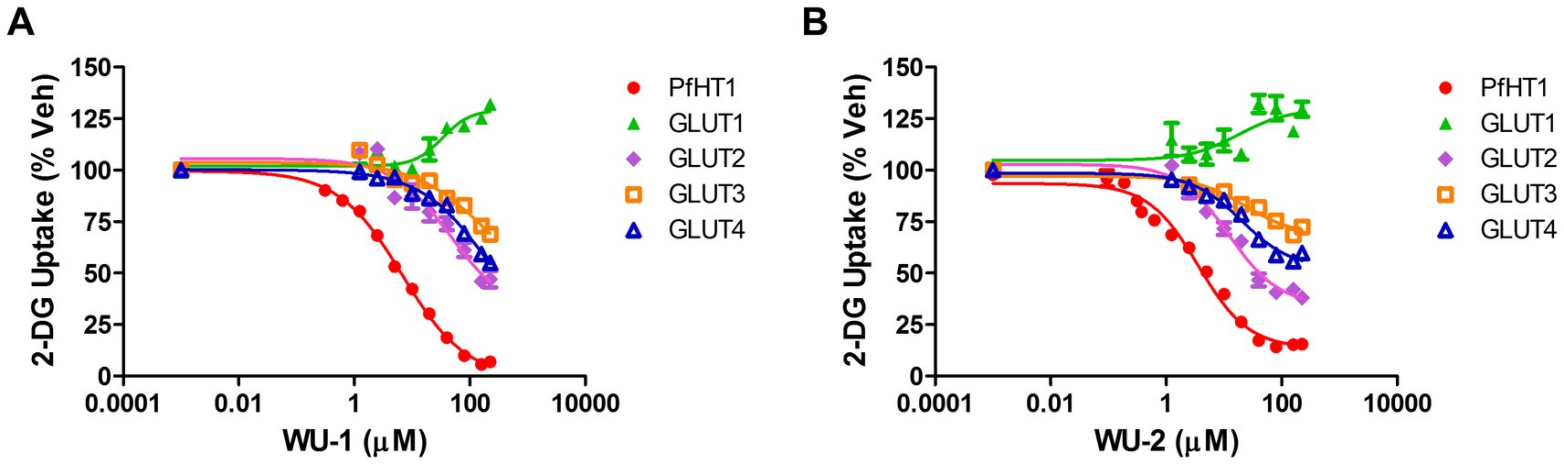
IC_50_ determination of glucose uptake by HEK293 cells overexpressing human class I orthologues. 2-DG uptake in response to increasing WU-1 (A) or WU-2 (B) concentrations in HEK293 cells expressing the indicated GLUT protein. IC_50_s were calculated using nonlinear regression analysis using GraphPad Prism 6.0 and are tabulated in Table 1. Uptake data are expressed as means ± SEMs of three independent experiments performed in triplicate.

To further characterize the specificity of WU-1 and WU-2 for PfHT over the human class I GLUT orthologues, we performed 2-DG uptake IC_50_ determinations on GLUTs 1–4 (Fig 3). Both WU-1 and WU-2 exhibited similar potencies for PfHT at 5.8 ± 0.6 μM and 4.2 ± 0.8 μM, respectively (Table 1). Due to solubility issues, we were unable to test these compounds at concentrations higher than 225 μM. As a result, the IC_50_ curves shown for WU-1 in Fig 3A are lacking data points that would comprise the lower asymptote of the curve that is necessary to accurately determine an IC_50_. Thus while WU-1 is selective for PfHT vs. the class I GLUTs, WU-2 is less selective, inhibiting GLUTs 2 and -4, albeit with less efficacy than PfHT as demonstrated by the shallowness of the curves (Fig 3 and Table 1).

**Table 1:** IC_50_ concentrations and maximal inhibition for WU-1 and WU-2 for class I GLUT overexpressing cells.

Cell Line	WU-1 IC_50_ (μM)	Maximum Inhibition (%) (%)	WU-2 IC_50_ (μM)	Maximum Inhibition (%)
PfHT	5.8 ± 0.6	93.2	4.2 ± 0.8	84.4
GLUT1	n.i.	n.a.	n.i.	n.a.
GLUT2	>50	n.a.	15.9 ± 2.5	62.0
GLUT3	>100	n.a.	>50	n.a.
GLUT4	>100	n.a.	18.5 ± 3.5	40.0

IC_50_ determinations for 2-DG uptake by PfHT and GLUTs 1–4 were determined as described in Materials and Methods. n.i = Not inhibited; n.a. = not available.

**Fig 4:**
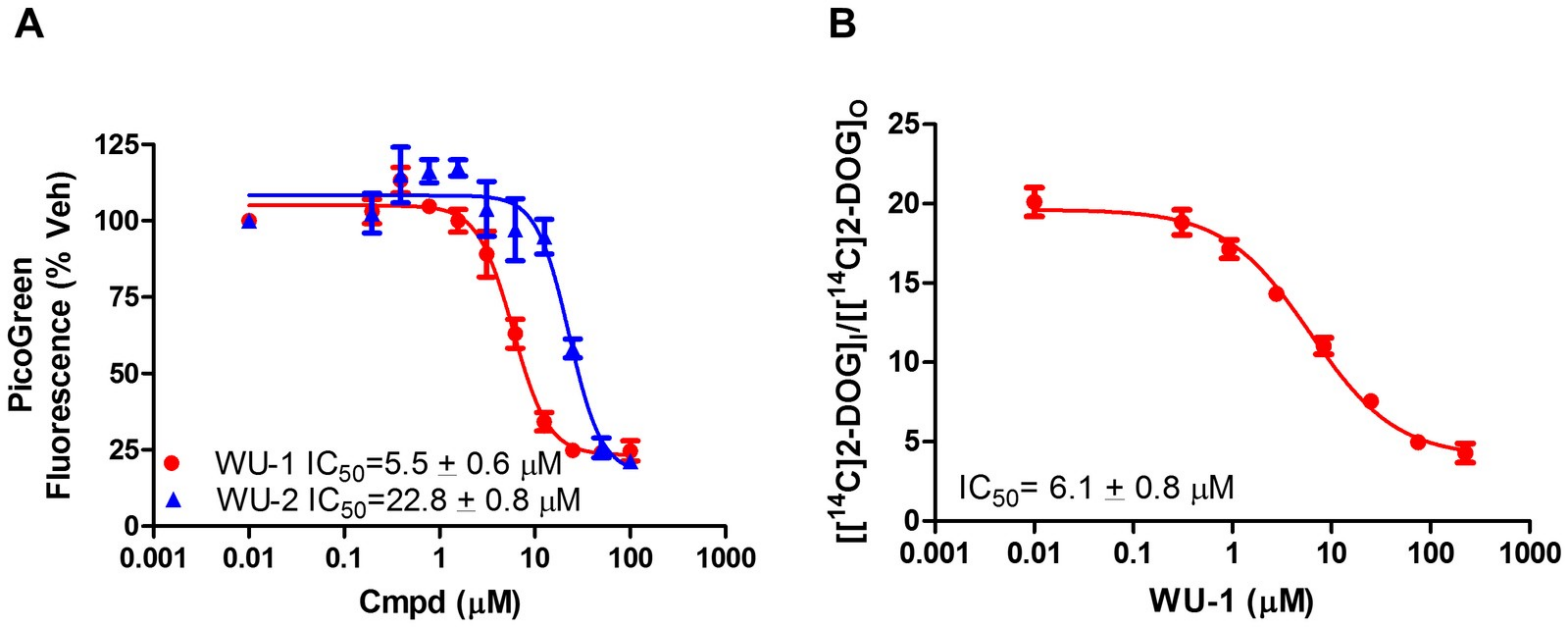
Inhibition of growth and IC_50_ determination of glucose uptake by isolated *P*. *falciparum* parasites. A) IC_50_s for inhibition of growth of *P*. *falciparum* strain 3D7 intraerythrocytic forms by each compound were determined via a growth inhibition assay as described in Materials and Methods. Data are expressed as means ± SEMs of three independent experiments performed in duplicate. B) Uptake of 2-DG by isolated *P*. *falciparum* trophozoites at increasing concentrations of WU-1. Distribution ratios (i.e., the ratio of intracellular concentration of radiolabel relative to the extracellular concentration) were calculated as described previously [23, 50]. IC_50_s were calculated using nonlinear regression analysis (GraphPad Prism 6.0). Uptake data are expressed as means ± SEMs of three independent experiments performed in triplicate.

**Fig 5:**
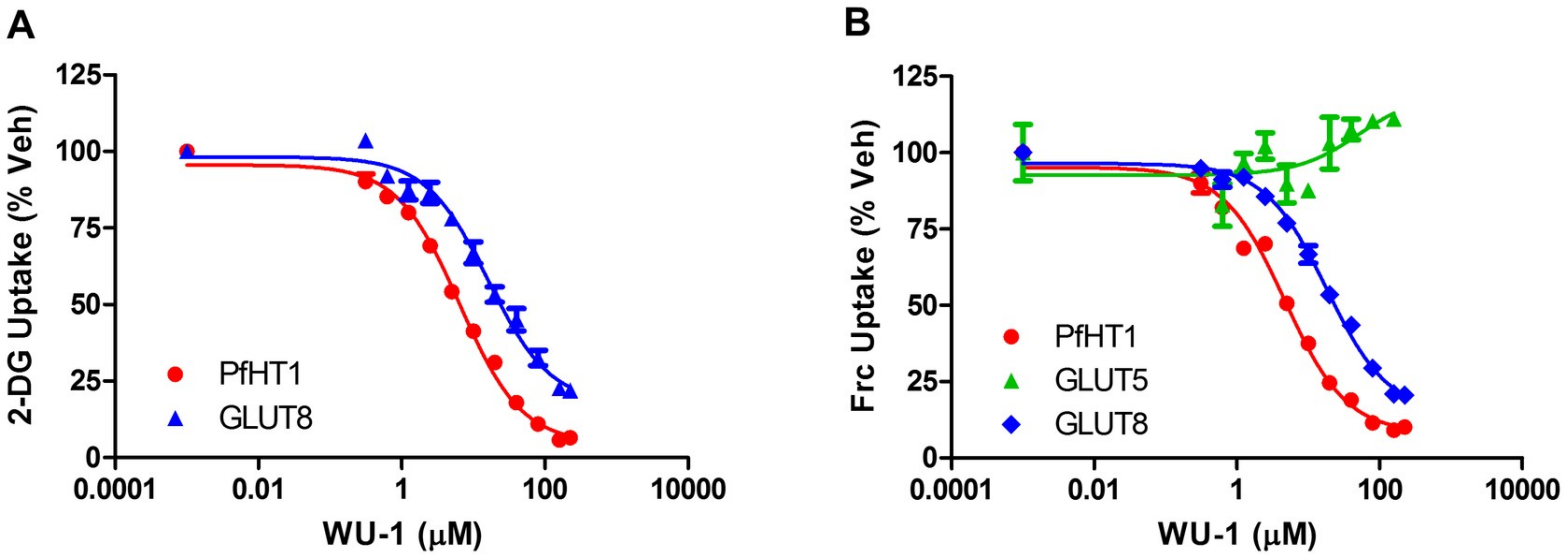
IC_50_ determination of hexose uptake by HEK293 cells overexpressing representative human class II and III orthologues. A) Uptake of 2-DG by HEK293 cells overexpressing either PfHT or class III GLUT transporter, hGLUT8. B) Uptake of [^3^H]-D-fructose (Frc) in HEK293 cells overexpressing hGLUT8, or PfHT or class II transporter, GLUT5. Uptake data are expressed as means ± SEMs of three independent experiments performed in triplicate. IC_50_s were calculated using nonlinear regression analysis (GraphPad Prism 6.0) and are tabulated in Table 2.

We next tested whether WU-1 and WU-2 inhibit asexual *P*. *falciparum* parasite growth in the *in vitro P*. *falciparum* growth inhibition assay [22]. As shown in Fig 4A, the EC_50_ curves for WU-1 and WU-2 inhibition of parasite growth were 5.5 ± 0.6 μM and 22.8 ± 0.8 μM, respectively. Since the compounds need to traverse the erythrocyte membrane in order to reach the parasite, we hypothesize that the right-shift in IC_50_ for WU-2 on the growth inhibition curve for *P*. *falciparum* is due to poor ADME (absorption, distribution, metabolism and excretion) characteristics for this compound. For this reason, as well as a diminished GLUT selectivity vs. WU-1, we focused on compound WU-1 for further characterization. The IC_50_ for 2-DG uptake by isolated freed parasites for WU-1 (Fig 4B) was similar to the EC_50_ growth inhibition curve for *P*. *falciparum* (6.1 ± 0.8 μM and 5.5 ± 0.6 μM, respectively). These data demonstrate inhibition of PfHT in a cellular context and support an antiparasitic mechanism-of-action of WU-1 through inhibition of glucose uptake by PfHT. In addition to the class I GLUTs, we were also interested in determining whether WU-1 was selective for PfHT over the human class II and class III GLUTs. Using HEK293 cells overexpressing GLUT5 or GLUT8 as representatives of class II and class III GLUTs, respectively, we determined the WU-1 IC_50_ for hexose uptake by these transporters. PfHT and GLUT8 transport both glucose and fructose while GLUT5 transports only fructose. The IC_50_ for PfHT was similar for 2-DG and D-fructose uptake (5.8 ± 0.6 μM vs 5.0 ± 0.7 μM, Fig 5 and Table 2). WU-1 failed to inhibit fructose uptake by class II GLUT5 (Fig 5B) and exhibited selectivity for PfHT over GLUT8 for both 2-DG and D-fructose substrates (Fig 5 and Table 2). Taken together, these data suggest that WU-1 exhibits selectivity for PfHT over the human class I, II and III GLUTs.

**Table 2:** IC_50_ concentrations and selectivity indices for WU-1 in GLUT5 and GLUT8 overexpressing cells.

Cell Line	2-DG (μM)	Selectivity Index	Frc (μM)	Selectivity Index
PfHT1	5.8 ± 0.6	1.0	5.0 ± 0.7	1.0
GLUT5	n.d.	n.d.	n.i.	n.i.
GLUT8	25.0 ± 7.5	4.3	22.0 ± 1.2	4.4

IC_50_ determinations for 2-DG and [^3^H] D-fructose uptake by PfHT-, GLUT5- and GLUT8-over-expressing cells were determined as described in Materials and Methods. The selectivity indices for PfHT over the individual GLUT proteins was calculated using these numbers. n.d. = not determined; n.i = Not inhibited.

### WU-1 is a non-competitive inhibitor of PfHT-mediated fructose uptake

**Fig 6:**
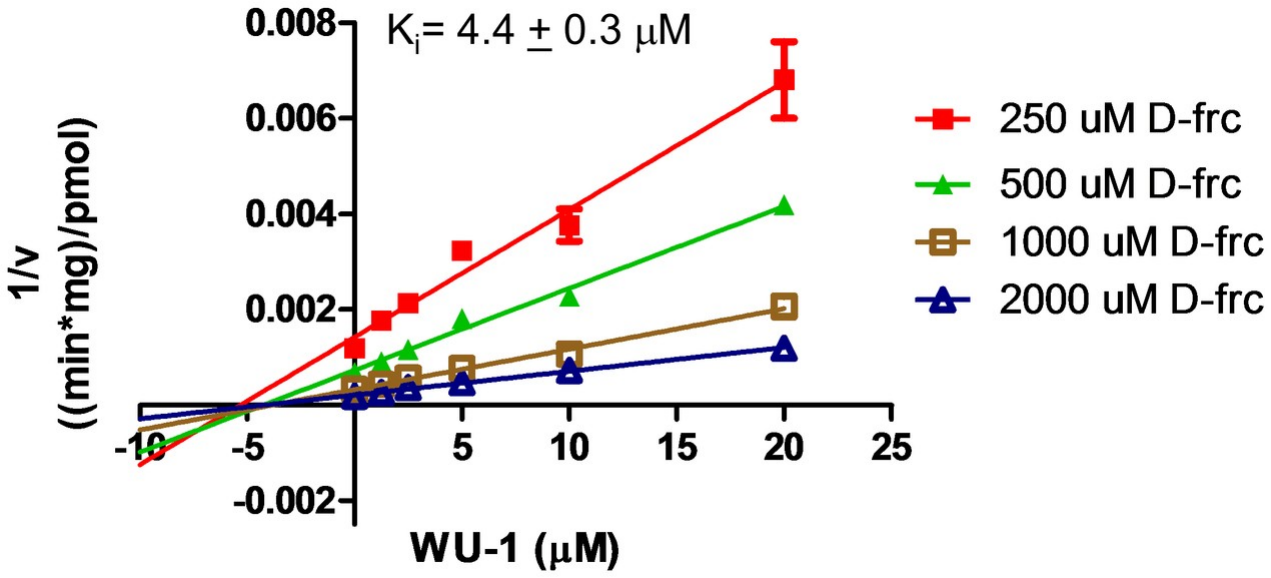
C-1 is a non-competitive inhibitor of PfHT. HEK293PfHT cells were incubated with increasing concentrations of [^3^H]-D-fructose (Frc). Data were fit by non-linear regression analysis using GraphPad Prism 6.0 software. Uptake data are expressed as means ± SEMs (n = 3). Dixon plot shows WU-1 is a noncompetitive inhibitor with a K_i_ of 4.4 ± 0.3 μM.

Kinetic analysis of hexose transport was performed to gain insight into the mode of action of WU-1 on PfHT. In order to obtain the clearest results, these experiments were carried out with fructose to eliminate any contribution from endogenous transporters. Fructose transport in these cell lines was over 20 fold above untransfected HEK293 cells (S2 Fig). PfHT is the only fructose transporter in PfHT-overexpressing HEK 293 cells. As shown in the Dixon Plot (Fig 6), the linear lines derived using different fructose concentrations intersect near the horizontal axis indicating that WU-1 is a non-competitive inhibitor of PfHT for zero-trans fructose uptake. The K_i_ of 4.4 ± 0.3 μM, calculated from the horizontal intercepts, is consistent with the IC_50_ for inhibition of fructose uptake in HEK293-PfHT cells (5.0 ± 0.7 μM, Table 2).

### WU-1 directly inhibits both the binding of the glucose analogue (ATB-BMPA) and glucose uptake in PfHT liposomes

**Fig 7:**
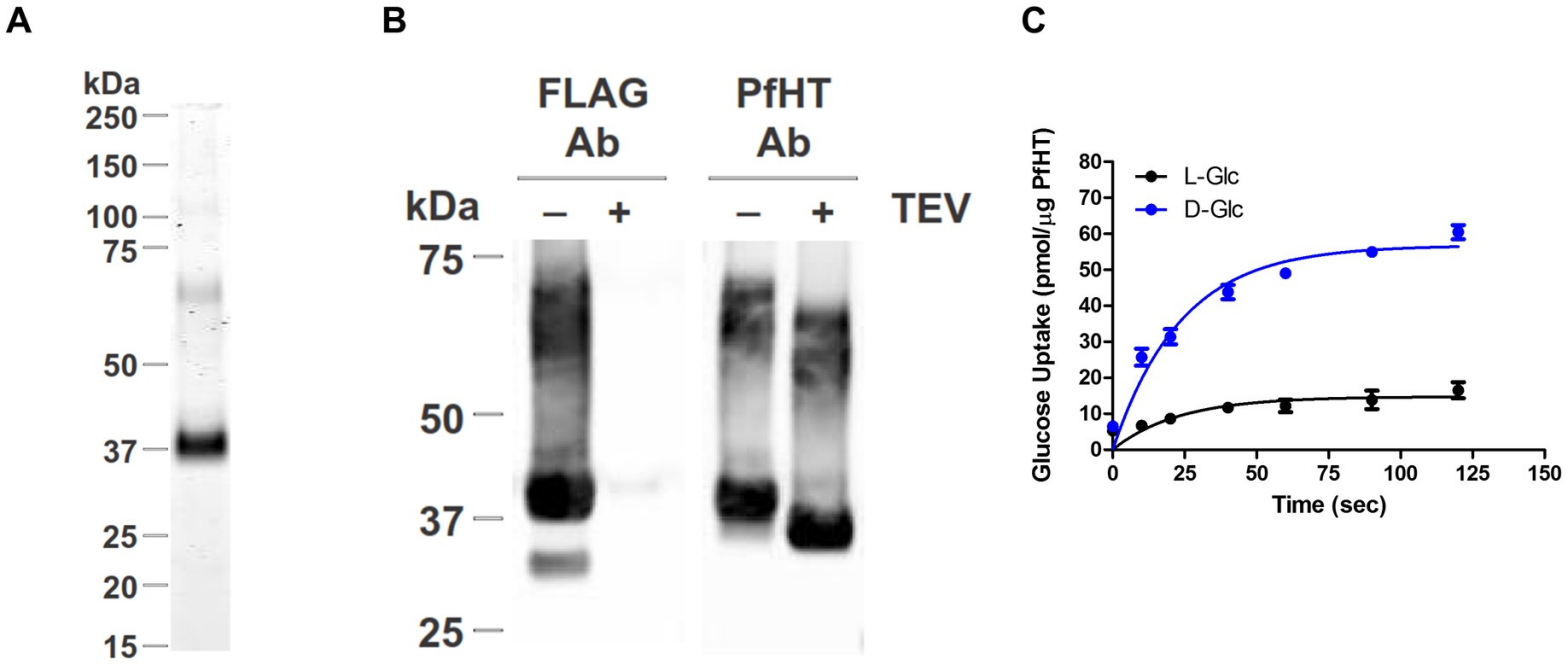
PfHT mediated uptake of [^3^H]-D-glucose and [^3^H]-L-glucose into liposomes. Purified PfHT protein (± FLAG tag) was reconstituted into eggPC/POPA/POPE (70:15:15) liposomes. A) SDS-PAGE of PfHT liposomes stained with Blue Bandit protein stain. B) Immunoblot analyses of PfHT liposomes using an anti-FLAG antibody (Ab) and a C-terminal PfHT specific antibody. (±) TEV indicates whether the FLAG tag of purified FTPfHT protein was cleaved off with TEV protease prior to the reconstitution into liposomes. C. Saturable, zero-trans uptake of [^3^H]-D-glucose into PfHT (-FLAG) liposomes (blue) compared to nonspecific uptake of [^3^H]-L-glucose (black). Uptake data were fitted using nonlinear regression analysis (GraphPad 6.0). Data are expressed as means ± SEMs (n = 3).

To verify that WU-1 inhibits PfHT-mediated glucose transport by directly interacting with PfHT, we engineered HEK293 cells stably overexpressing PfHT containing an N-terminal FLAG tag that can be cleaved using tobacco etch virus (TEV) protease. Using a protocol we developed to reconstitute GLUT4 into eggPC/POPA/POPE (70:15:15) liposomes [26, 27], we were able to purify FLAG-tagged PfHT to ~95% homogeneity using an M2 anti-FLAG affinity gel (Fig 7A). An immunoblot of PfHT liposomes using a FLAG-tag specific antibody revealed a major band ~38–40 kDa and two diffuse minor bands ~65–70 kDa (Fig 7B) all of which disappeared with cleavage of the FLAG tag via TEV protease. The same immunoblot bands appeared using a PfHT specific antibody that recognizes a peptide in the C-terminus (Fig 7B). TEV cleavage increased the mobility of both the major and minor bands. Our interpretation of the Western blot data is that the 38–40 kDa band represents full length PfHT and the 65–70 kDa band is likely a PfHT dimer that is commonly found in SDS gels of other glucose transporters (experimental observation). Whether the dimer is of physiological importance as has been demonstrated for GLUT1 [30] or just a gel artifact of solubilizing a 12-helical membrane protein in detergent is unknown. The same molecular bands were observed when purified PfHT was run on a gel and then stained with Blue Bandit protein stain (Fig 7A). To test the functional activity of PfHT in liposomes, we measured zero-trans specific uptake of [^3^H]-D-glucose and nonspecific [^3^H]-L-glucose (Fig 7C). Uptake was saturable with the incorporation of D-glucose ~4-fold higher than that of L-glucose indicating successful incorporation of functional PfHT protein into liposomes.

**Fig 8:**
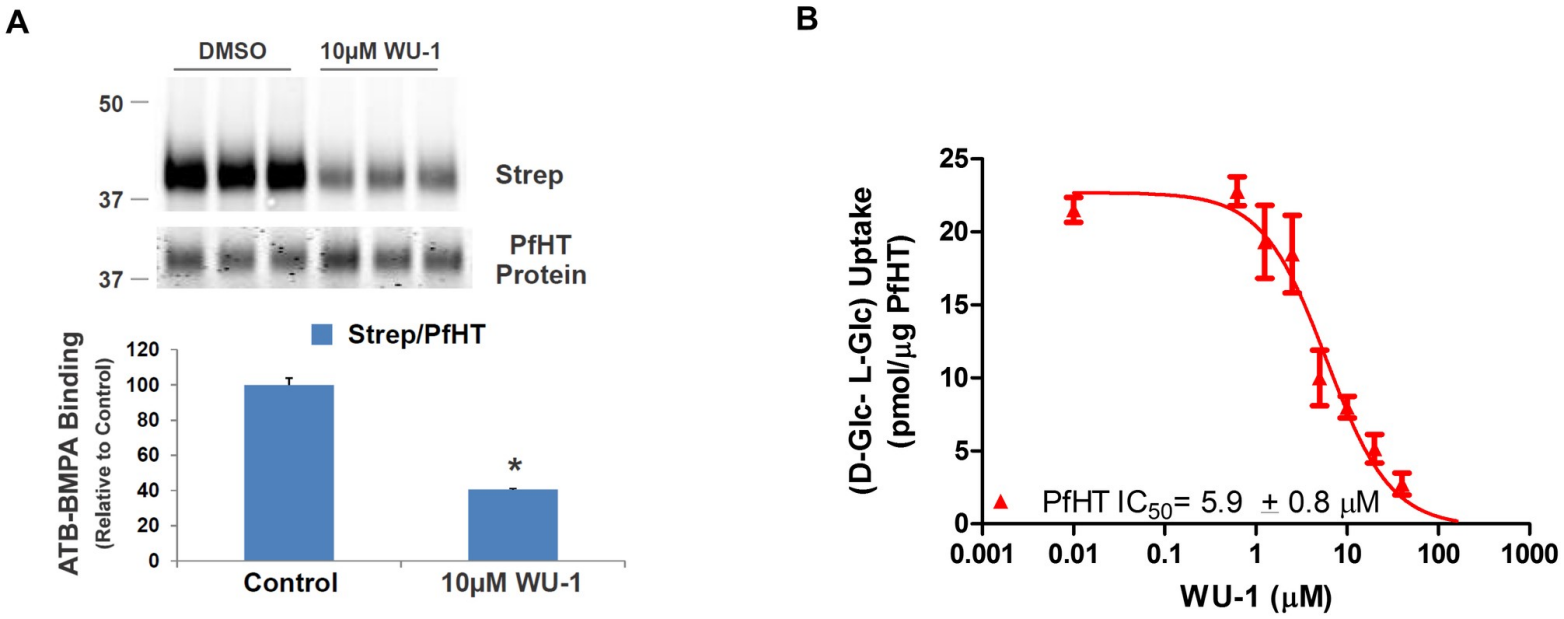
WU-1 directly inhibits both the binding of the glucose analogue (ATB-BMPA) to PfHT and PfHT transport activity. A) Biotinylated ATB-BMPA, a bis-mannose containing photolabel, binds to the glucose binding site of PfHT. Liposomes containing 0.65 μg PfHT were preincubated with ± 10 μM WU-1 prior to labeling with biotinylated ATB-BMPA. PfHT bound with biotinylated ATB-BMPA was analyzed by immunoblot analysis using a fluorescently labeled streptavidin (Strep). The strep signal was normalized to the amount of PfHT protein. Data are expressed as the means ± SEM., n = 3; (*), p<0.05 vs. vehicle control as determined by the paired Student’s t test. B) WU-1 inhibits the specific uptake ([^3^H]-D-glucose minus ([^3^H]-L-glucose) into PfHT-containing liposomes. Different concentrations of WU-1 were added to the liposomes 20 min prior to the initiation of the transport reaction. Uptake (quenched after 50 sec) was normalized to the amount of PfHT in the liposomes. Data were fit by nonlinear regression analysis using GraphPad Prism 6.0 software to calculate the IC_50_ for WU-1. Data are expressed as mean ± SEM of three independent experiments.

Next, we used an ATB-BMPA photolabel binding assay [24, 31] to analyze ligand binding and competition with WU-1 in PfHT-containing liposomes. Biotinylated ATB-BMPA contains the glucose analogue bis-mannose and a photolabel that can irreversibly bind to the glucose binding site of class I transporters [32]. Binding of the biotinylated photolabel to PfHT, monitored by immunoblot analysis using a fluorescently labeled streptavidin, was ~60% less in the presence of 10 μM WU-1 (Fig 8A), demonstrating that WU-1 binds directly to PfHT.

PfHT liposomes were then used to test the potency of WU-1 to inhibit PfHT-mediated glucose uptake. WU-1 inhibited zero-trans specific uptake (D-glucose minus L-glucose) with an IC_50_ of 5.9 ± 0.8 μM (Fig 8B). Due to the consistency of the IC_50s_ in liposomes (5.9 ± 0.8 μM), in HEK293-PfHT cells (5.8 ± 0.6 μM), in freed parasites (6.1 ± 0.8 μM), coupled with the EC_50_ for growth inhibition in *P*. *falciparum* (5.5 ± 0.6 μM), we conclude that WU-1 kills the parasite by directly inhibiting PfHT.

### Insertion of an N-terminal FLAG tag to PfHT affects the ability of endofacial and exofacial ligands to inhibit glucose transport

**Fig 9:**
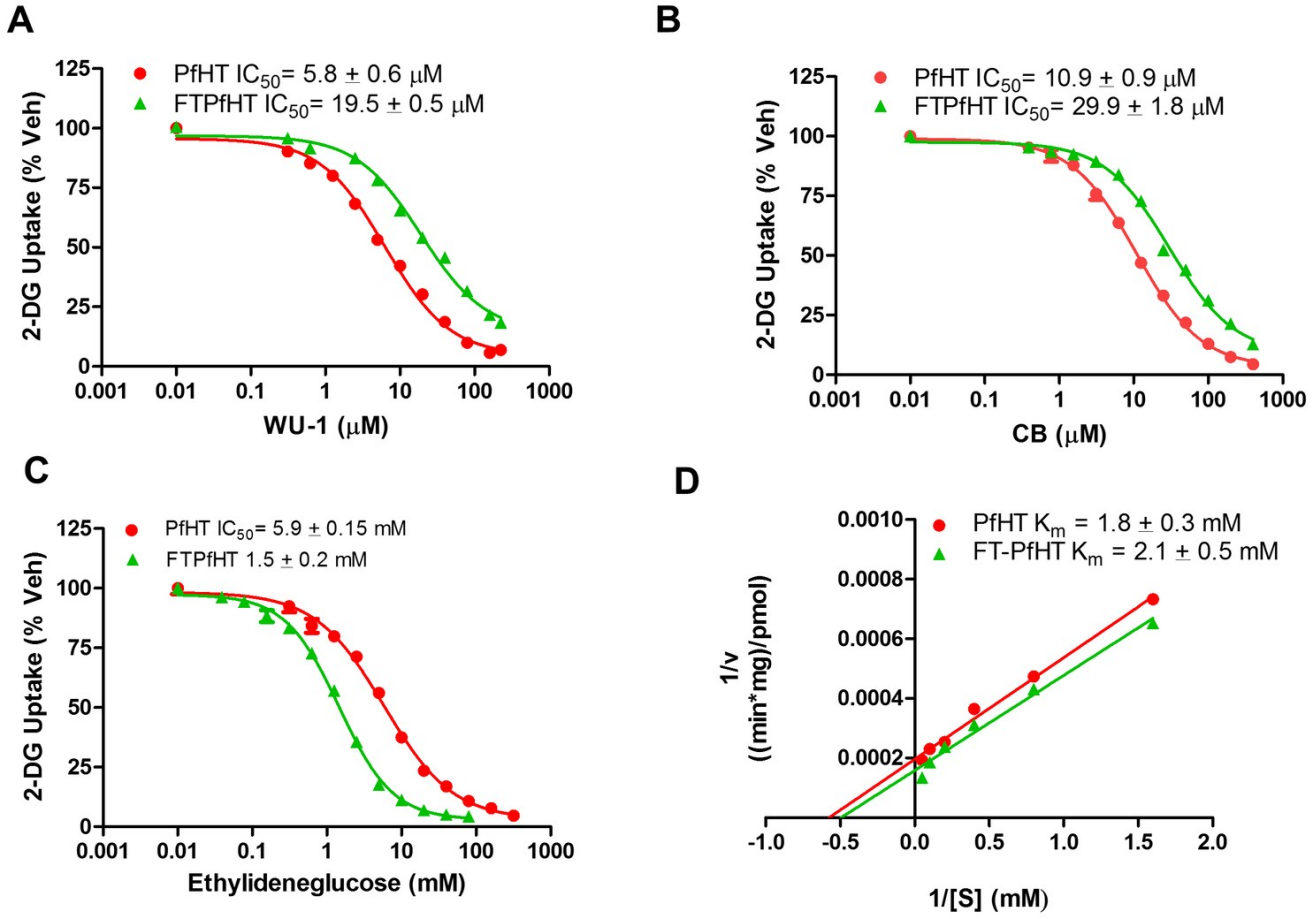
Effect of the FLAG tag on the inhibition of PfHT-mediated glucose uptake by WU-1, CB, and ethylideneglucose. A) Inhibition of 2-DG uptake by WU-1 in HEK293 cells overexpressing PfHT and FTPfHT. B) Inhibition of 2-DG uptake by the endofacial ligand cytochalasin B (CB) in HEK293 cells overexpressing PfHT and FT-PfHT. C) Inhibition of 2-DG uptake by the exofacial ligand ethylideneglucose in HEK293 cells overexpressing PfHT and FTPfHT. Data (Panels A-C) were fit by nonlinear regression analysis using GraphPad Prism 6.0 software to calculate the IC_50s_. Data are expressed as means ± SEMs of three independent experiments performed in triplicate. D) Kinetic analysis showed that the FLAG-tag had little effect on the K_m_. 2-DG uptake (1 min) were measured in HEK293 cells overexpressing PfHT and FTPfHT at different 2-DG concentrations. K_m_ values were determined from nonlinear regression analysis using GraphPad Prism 6.0. Data are plotted in double-reciprocal form and expressed as the means ± SEMs of three independent experiments performed in triplicate.

As described above, a FLAG tag was attached to the amino terminus of PfHT in order to purify PfHT prior to its reconstitution into liposomes. To test whether the FLAG tag itself affected PfHT transporter activity, we measured the ability of WU-1 to inhibit radiolabeled 2-DG uptake in PfHT and FLAG-tag-PfHT (FTPfHT) overexpressing cells. The WU-1 IC_50_ value for FTPfHT was significantly right shifted compared to that for wild-type PfHT (19.5 ± 0.5 μM vs. 5.8 ± 0.6 μM, p< 0.01) indicating that WU-1 is less potent in inhibiting FLAG-tagged PfHT protein (Fig 9A). Similar results were found in liposomes, where the WU-1 IC_50_ value for FTPfHT was 13.2 ± 3.1 μM compared to 5.9 ± 0.8 μM for PfHT (S3 Fig). Although WU-1 is a non-competitive inhibitor of PfHT in terms of zero-trans influx (Fig 6), it remains possible that WU-1 is a competitive inhibitor for zero-trans efflux as has been shown for cytochalasin B (CB) [33] and for the HIV protease inhibitor indinavir [31]. Similar to all GLUTs that are inhibited by CB, residues Q282, W388, N411, and W412 (GLUT1 nomenclature) are found in PfHT [34]. When the endofacial ligand CB was used to inhibit glucose uptake in both cell lines, like WU-1, we found that CB was significantly less potent (right-shifted) in inhibiting FTPfHT than PfHT (IC_50_ = 29.9 ± 1.8 μM vs 10.9 ± 0.9 μM, p< 0.05, Fig 9B). Using the exofacial ligand 4,6-O-ethylidene-α-D-glucose, the opposite effect was observed. Ethylideneglucose was significantly more potent (left-shifted) in inhibiting FTPfHT-mediated glucose uptake compared to PfHT (IC_50_ = 1.5 ± 0.2 mM vs. 5.9 ± 0.15 mM, p < 0.001, Fig 9C). Kinetic analysis revealed that the K_m_ for FTPfHT (2.1 ± 0.5 mM) was not statistically different for that of PfHT (1.8 ± 0.3 mM) indicating that the transporter affinity for glucose was unaffected by the FLAG tag (Fig 9D). The K_m_ for PfHT is similar to that reported for PfHT in oocytes [35].

## Discussion

In this study, we have identified WU-1 as a small molecule inhibitor of the *P*. *falciparum* hexose transporter, PfHT. After improving the throughput of our previously reported 96-well cell-based assay for the screening of small molecules that inhibit glucose uptake by PfHT [15] to run in a 384-well format, we screened of the Maybridge HitFinder library of 14,399 drug-like compounds. WU-1 exhibited potency for PfHT inhibition in the low micromolar range, efficacy in inhibiting parasite growth and excellent selectivity against the human GLUT isoforms vs. the other confirmed hits identified in the screen. The strong correlation between the IC_50_ of WU-1 for PfHT-overexpressing HEK293 cells, EC_50_ for parasite growth inhibition and inhibition of glucose uptake in freed parasites suggests that PfHT inhibition is the mechanism of parasite death. That WU-1 is selective for PfHT over the human orthologues is important when considering potential safety issues in non-selectively inhibiting human GLUT transporters. GLUT1 is ubiquitously expressed in most tissues and is the key GLUT for glucose transport in the brain [36]. GLUT4 is the key glucose transporter in muscle and fat for the transport of glucose in response to insulin, the inhibition of which leads to insulin resistance [37]. Thus, maintaining selectivity for PfHT over the class I GLUTs including GLUTs 1 and 4 is essential for development of an effective and safe therapeutic. Importantly, WU-1 had excellent selectivity for PfHT over insulin-dependent GLUT4 (GLUT4 IC_50_> 100 μM). Although the functional significance of the class II and III GLUTs has been only partially elucidated, there is high potential that disruption of the functional activity of these transporters in humans would have significant metabolic effects [38]. Thus, it is advantageous that WU-1 exhibits selectivity for PfHT over the class III transporter GLUT8, and has no effect on GLUT5, a class II transporter.

An important aspect of drug development is determining how the identified hit(s) are inhibiting the target of interest. For PfHT, if the drug binding site overlaps with the substrate binding site this should aid in preventing drug resistance. Specifically, mutations that prevent drug binding would be more likely to also disrupt transporter function. In PfHT overexpressing HEK293 cells, we determined that WU-1 is a noncompetitive inhibitor of PfHT-mediated zero-trans hexose influx. As for other facilitative transport proteins that mediate bi-directional flux across cell membranes, this is the expected pattern of inhibition for compounds that act at the endofacial side of the glucose permeation pathway. Inhibitor binding to the contralateral membrane surface from the measured transport rate would not be expected to be displaced by substrate. This phenomenon is well established for CB and other known endofacial GLUT ligands [31, 39]. Further support for WU-1 endofacial binding is that the IC_50_s for WU-1 and CB were both right-shifted in FLAG-tagged PfHT compared to PfHT over-expressing cells whereas the exofacial ligand ethylideneglucose was left-shifted. Crystallization of the protein with the drug bound would definitively identify the drug binding site similar to what was achieved for GLUT1, CB, and two Phe amide-derived inhibitors [40, 41]. Using a cell line that overexpresses FLAG-tagged PfHT, we purified PfHT to over 95% and determined it was functional and able to transport D-glucose. Expression and purification of functional PfHT is a vital step in crystalizing the protein. Using our novel ATB-BMPA assay, WU-1 inhibited ATB-BMPA binding to PfHT liposomes indicating that WU-1 interacts directly with the transporter. This was further supported by data demonstrating that WU-1 inhibits glucose uptake in liposomes containing purified PfHT with an IC_50_ of 5.9 ± 0.8 μM, consistent with the IC_50_s determined in PfHT cells and freed parasites, and the EC_50_ for growth inhibition.

**Fig 10:**
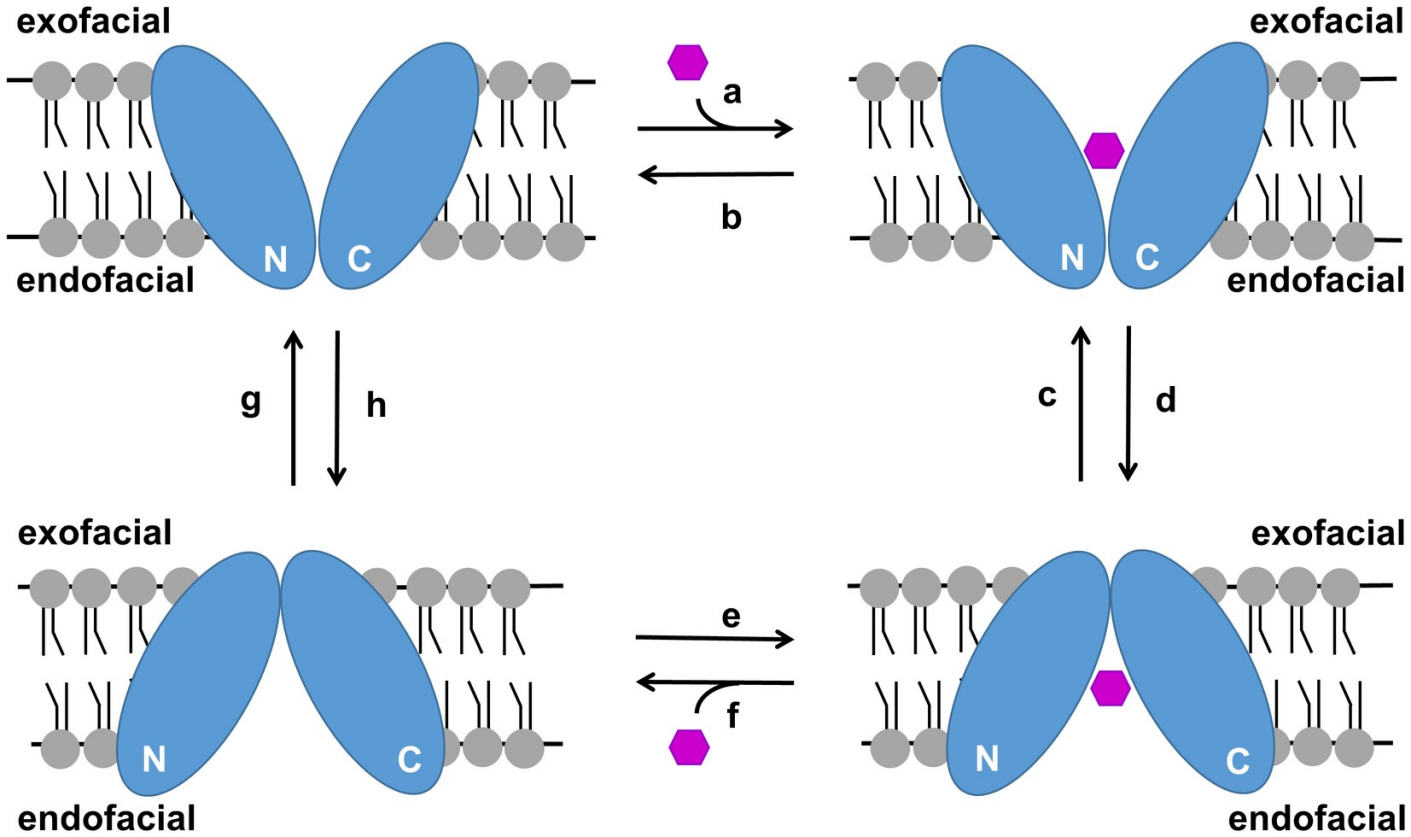
Simple carrier model illustrating the transport of glucose by an alternating access mechanism. Glucose can access the glucose binding site of PfHT from either the exofacial side or the endofacial side of the membrane but not simultaneously. N and C represent the N-terminal and C-terminal α-helical bundles of PfHT. Upon binding glucose, PfHT undergoes a series of conformational changes that allows the translocation of glucose through the central pore and its release from PfHT at the other side of the membrane. Transport is bidirectional and down the concentration gradient.

All glucose transporters are thought to transport glucose by an alternating access mechanism in which a centrally located glucose binding site is alternatingly accessed from either side of the membrane through a series of conformational changes in the protein (Fig 10). The GLUTs as well as PfHT are members of the major facilitator superfamily (MFS) of transporters which all possess a characteristic fold consisting of two symmetrical six transmembrane bundles. Recently published crystal structures of GLUT1, GLUT3, GLUT5, and XylE [42–46] indicate that the alternation between outward- and inward-facing conformations is achieved via a rocker-switch motion and by a gated-pore mechanism involving transmembrane helices 7 and 10. Transition from the inward-facing to the outward-facing conformation is catalyzed by the transient formation of salt bridges at the endofacial side of the transporter. These salt bridges are formed either between the cytoplasmic ends of several transmembrane helices or between intracellular and transmembrane helices. Interestingly, the FLAG tag (DYKDDDDK) which contains seven charged amino acids may also be capable of forming transient salt bridges with residues within PfHT. Our zero-trans influx data indicates that adding a FLAG tag to the N-terminus of PfHT decreases the potency of an endofacial ligand, CB, increases the potency of the exofacial ligand, ethylideneglucose, but has no effect on the K_m_. According to the recently published crystal structure of GLUT5, the cytoplasmic N-terminus is both facing and in close proximity to several regions involved in transient salt bridge formations in the outward-facing but not in the inward-facing conformation (Extended data Fig 7 of Reference [43]). Based on our experimental data and the published crystallographic data, we propose a model in which the FLAG tag, when attached to the N-terminus of PfHT, provides additional stabilization of the outward-facing conformation in the absence of substrate via transient salt bridges with residues found in PfHT. The actual tethering of the FLAG tag to the N-terminus of PfHT appears to be important to our experimental observations since the addition of excess FLAG peptide to PfHT liposomes had no effect on glucose uptake. In addition to the FLAG motif (DYKDDDDK), our engineered FLAG tag contains a TEV protease site (ENLYFQS) flanked on both sides by triple glycine linkers which may add crucial flexibility in salt bridge formations (see Materials and methods). Assuming a simple carrier model for PfHT, stabilization of the outward-facing conformation in a ligand empty transporter would occur by increasing the rate of step “g” relative to the rate of step “h” (Fig 10). Our hypothesis is also consistent with data reported for GLUT1 that was locked in the outward-facing conformation by an Y293I substitution [47]. This mutation decreased the K_i_ for ethylideneglucose > 2-fold, increased the K_i_ for CB >100-fold, and had little effect on the K_m_. In contrast to our results, 2-DG uptake was greatly impaired presumably since GLUT1 was actually locked in one conformation, while FTPfHT is still active.

Our identification and initial characterization of a protein modification that appears to alter the conformational dynamics of PfHT has potential clinical relevance. Specifically, stabilization of the outward-facing conformation should increase the overall rate of the glucose transport cycle since there would be more ligand empty transporters primed to bind and transport extracellular glucose molecules (Fig 10). Although this would not be a desirable effect in efforts to treat malaria, our FLAG tag salt bridge hypothesis could apply to other mammalian GLUTs where increased activity would be of benefit. For example, augmentation of GLUT1 or GLUT4 intrinsic activity would be advantageous in patients with diabetes mellitus.

As with previously identified small molecule PfHT inhibitors, significant probing of identified chemical scaffolds via medicinal chemistry will be required to develop these newly identified hit compounds to the stage of lead optimization. The emergence of an expanding list of candidates greatly improves the likelihood of success in this endeavor. An advantage of the Maybridge HitFinder collection is the availability of a large set of “building block” analogs to facilitate this process.

In addition to significantly expanding the list of chemical scaffolds that can be used in the development of new anti-malarial drugs possessing optimal characteristics of efficacy, safety and durability against the development of resistance, the paradigm of targeting microbial hexose transport has significant potential for broader application. This includes other parasites such as *Leishmania* [48] and other pathogenic microbes that possess structurally similar and functionally essential hexose transporters [49]. While homologous to mammalian transporters, our current study demonstrates that selective targeting of parasite transporters over human orthologues can be achieved.

## Supporting information

S1 Fig Transcriptional characterization of GLUT family members in hGLUT5- and hGLUT8-overexpressing HEK293-FLIP cells. Copies of transcript per nanogram of cDNA for each glucose transporter SLC2A family member in: A) overexpression of hGLUT5 in HEK293-flip cells, B) overexpression of hGLUT8 in HEK293-FLIP cells in which native hGLUT1 expression was knocked down by siRNA as described in Materials and Methods.
(TIF)

S2 Fig Functional activity of HEK293 cell lines. Uptake of (A) radiolabeled 2-DG and (B) radiolabeled fructose in cell lines over-expressing the indicated hexose transporter. Data are shown as transport activity relative to untransfected HEK293 cells ± SEM of three determinations per cell line. The red line represents relative uptake in untransfected HEK293 cells.
(TIF)

S3 Fig WU-1 is less potent in inhibiting FTPfHT transporter activity in reconstituted liposomes. WU-1 inhibits the specific uptake ([^3^H]-D-glucose minus ([^3^H]-L-glucose) into FTPfHT-containing liposomes. Different concentrations of WU-1 were added to the liposomes 20 min prior to the initiation of the transport reaction. Uptake (quenched after 50 sec) was normalized to the amount of FTPfHT in the liposomes. Data were fit by nonlinear regression analysis using GraphPad Prism 6.0 software to calculate the IC_50_ for WU-1. Data are expressed as mean ± SEM of three independent experiments.
(TIF)

S1 File RAW data for PLOS ONE. File contains minimal data set used to reach the conclusions drawn in the manuscript including the values behind the means, standard deviations and other measures reported, the values used to build graphs, and the points extracted from images for analysis.
(XLSX)
