# Supplementary figures and images for: Identification of druggable small molecule antagonists of the Plasmodium falciparum hexose transporter PfHT and assessment of ligand access to the glucose permeation pathway via FLAG-mediated protein engineering
Source: PLoS One. 2019 May 9;14(5):e0216457. doi: 10.1371/journal.pone.0216457 (PMC6508677; doi:10.1371/journal.pone.0216457)

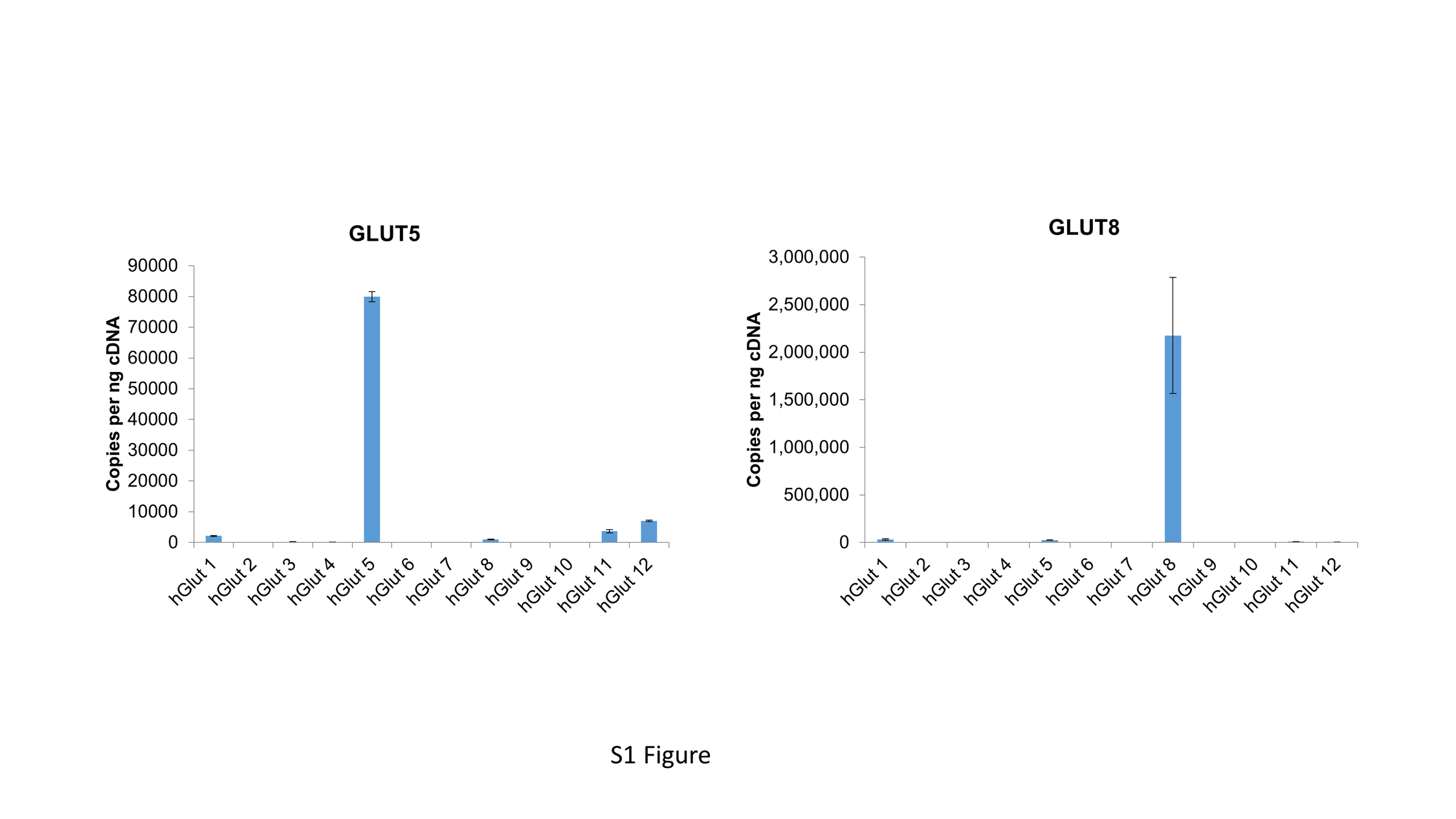

Supplement: S1 Fig — Copies of transcript per nanogram of cDNA for each glucose transporter SLC2A family member in: A) overexpression of hGLUT5 in HEK293-flip cells, B) overexpression of hGLUT8 in HEK293-FLIP cells in which native hGLUT1 expression was knocked down by siRNA as described in Materials and Methods. (TIF) [file pone.0216457.s001.tif]

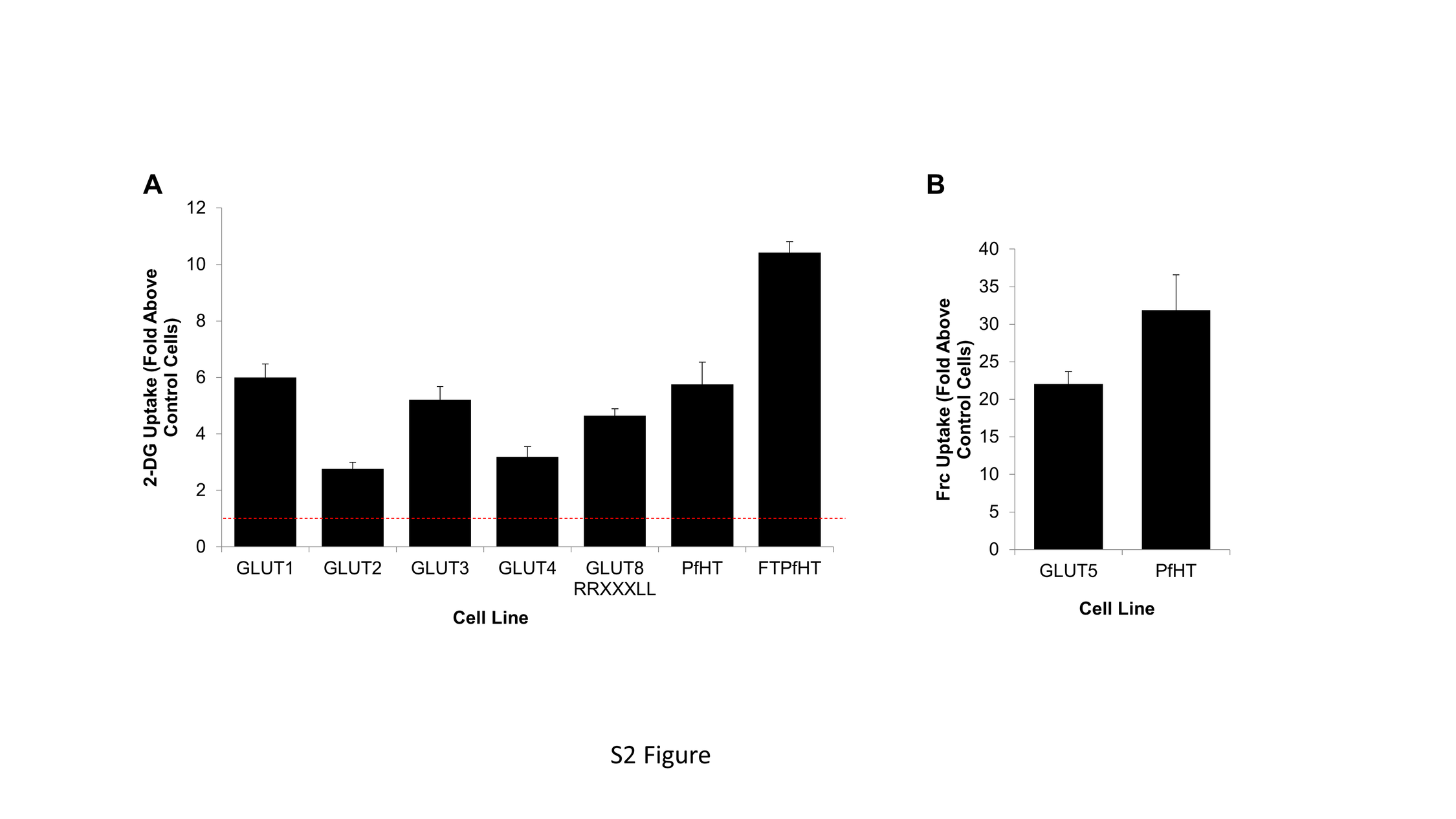

Supplement: S2 Fig — Uptake of (A) radiolabeled 2-DG and (B) radiolabeled fructose in cell lines over-expressing the indicated hexose transporter. Data are shown as transport activity relative to untransfected HEK293 cells ± SEM of three determinations per cell line. The red line represents relative uptake in untransfected HEK293 cells. (TIF) [file pone.0216457.s002.tif]

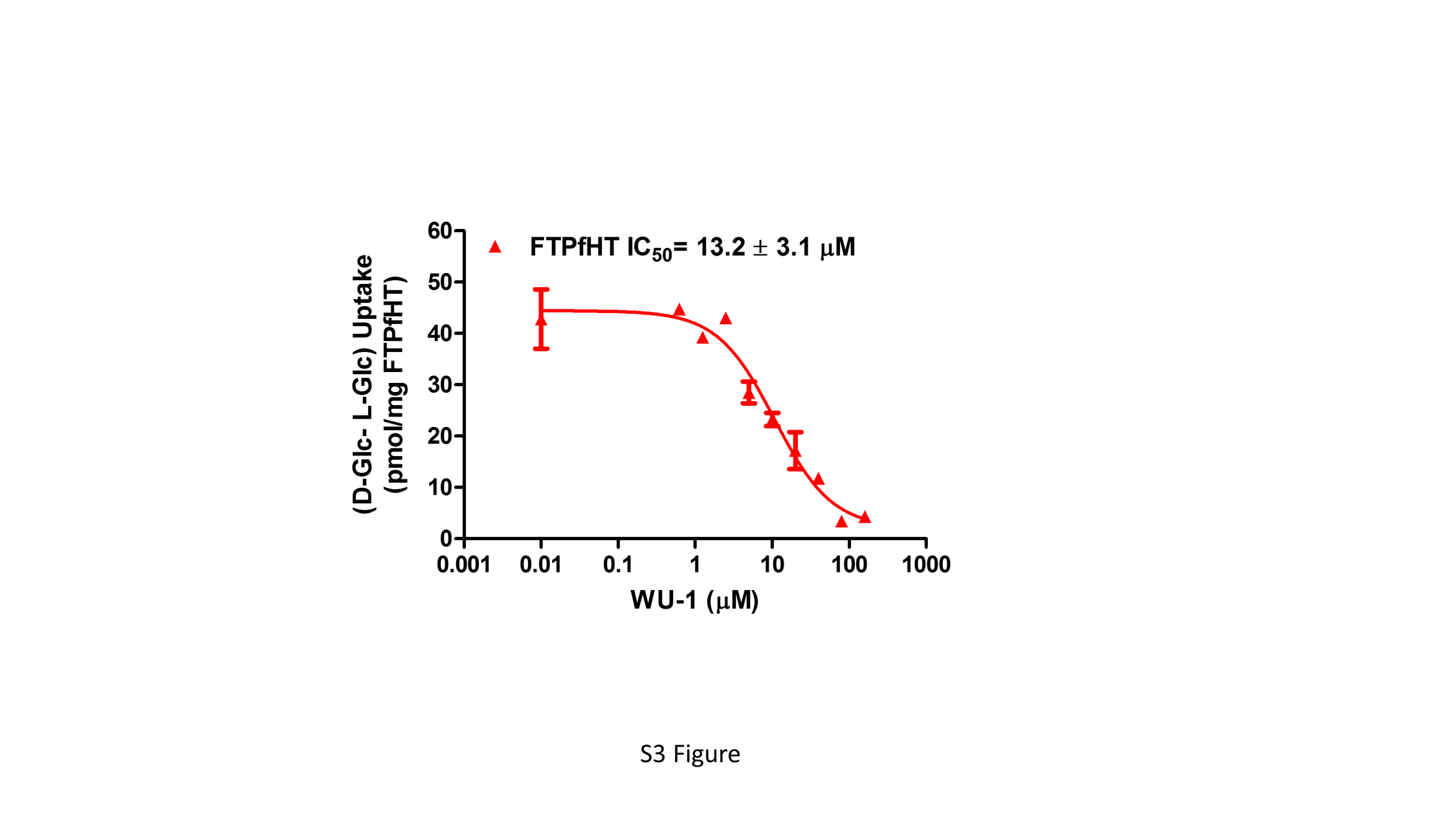

Supplement: S3 Fig — WU-1 inhibits the specific uptake ([3H]-D-glucose minus ([3H]-L-glucose) into FTPfHT-containing liposomes. Different concentrations of WU-1 were added to the liposomes 20 min prior to the initiation of the transport reaction. Uptake (quenched after 50 sec) was normalized to the amount of FTPfHT in the liposomes. Data were fit by nonlinear regression analysis using GraphPad Prism 6.0 software to calculate the IC50 for WU-1. Data are expressed as mean ± SEM of three independent experiments. (TIF) [file pone.0216457.s003.tif]
